# Bio-Based Poly(lactic acid)/Poly(butylene sebacate) Blends with Improved Toughness

**DOI:** 10.3390/polym14193998

**Published:** 2022-09-24

**Authors:** Adriana Nicoleta Frone, Marius Stelian Popa, Cătălina Diana Uşurelu, Denis Mihaela Panaitescu, Augusta Raluca Gabor, Cristian Andi Nicolae, Monica Florentina Raduly, Anamaria Zaharia, Elvira Alexandrescu

**Affiliations:** National Institute for Research and Development in Chemistry and Petrochemistry—ICECHIM, 202 Splaiul Independentei, 060021 Bucharest, Romania

**Keywords:** poly(butylene sebacate), biopolymer blends, thermal analysis, dynamic mechanical analysis

## Abstract

A series of poly(butylene sebacate) (PBSe) aliphatic polyesters were successfully synthesized by the melt polycondensation of sebacic acid (Se) and 1,4-butanediol (BDO), two monomers manufactured on an industrial scale from biomass. The number average molecular weight (M_n_) in the range from 6116 to 10,779 g/mol and the glass transition temperature (*T_g_*) of the PBSe polyesters were tuned by adjusting the feed ratio between the two monomers. Polylactic acid (PLA)/PBSe blends with PBSe concentrations between 2.5 to 20 wt% were obtained by melt compounding. For the first time, PBSe’s effect on the flexibility and toughness of PLA was studied. As shown by the torque and melt flow index (MFI) values, the addition of PBSe endowed PLA with both enhanced melt processability and flexibility. The tensile tests and thermogravimetric analysis showed that PLA/PBSe blends containing 20 wt% PBSe obtained using a BDO molar excess of 50% reached an increase in elongation at break from 2.9 to 108%, with a negligible decrease in Young’s modulus from 2186 MPa to 1843 MPa, and a slight decrease in thermal performances. These results demonstrated the plasticizing efficiency of the synthesized bio-derived polyesters in overcoming PLA’s brittleness. Moreover, the tunable properties of the resulting PBSe can be of great industrial interest in the context of circular bioeconomy.

## 1. Introduction

The persistent consumption of the already scarce global oil reserves and the increasing public awareness of the environmental problems caused by the improper management of plastic waste have encouraged the development of bio-based polymers as alternatives for the conventional non-biodegradable petroleum-based plastics. A very attractive substitute for the oil-based plastics is polylactic acid (PLA), an aliphatic polyester [1] whose synthesis is currently achieved by two main routes: the ring-opening polymerization (ROP) of the *L*-lactide dimer in the presence of a metal-based or organic catalyst, and the direct polycondensation of lactic acid [2]. Of the two methods, ROP is preferred, since it allows for the obtaining of PLA with high molecular weights in a shorter time [3], as well as good control over the molecular weight and polydispersity of the resulting polymer [4]. Regardless of the synthesis route, a great benefit is the possibility of obtaining the starting monomer—lactic acid—by the bacterial fermentation of sugars from renewable sources, such as corn, potato, wheat, rice, sugarcane, sugar beet pulp [5], or agricultural waste [6]. The most appealing characteristics of PLA reside in its origin from renewable sources, as well as its biodegradability, biocompatibility, and non-toxicity [7]. Additionally, its high tensile strength and elastic modulus, which are comparable to those of conventional oil-based polymers [8], and the possibility of processing PLA using all techniques characteristic of petroleum-based polymers [9], make PLA one of the most in-demand bioplastics on the market [10]. Today, PLA is used in a wide range of applications, from disposable packaging and food service ware [11]; to implants, drug delivery systems, scaffolds, sutures, wound dressings, and coatings in biomedicine [12]; toys and home appliances [13]; textiles [11]; and mulch films in agriculture [14]. Consequently, the global production of PLA is expected to grow from the almost 200,000 tons/year reported in 2019, to 370,000 tons/year by 2023 [15]. Nevertheless, the use of PLA on a larger scale is restrained due to its unsatisfactory properties, such as its inherent brittleness, low elongation at break, poor impact strength, low thermal stability, low heat distortion temperature, narrow processing window, and slow crystallization rate [16]. To increase the flexibility, ductility, toughness, and processability of PLA, several methods have been applied, plasticization, copolymerization, and physical blending being some of the most popular choices [17]. In order not to compromise the bio-based origin of PLA, in the last two decades, researchers have focused on finding effective agents for improving the flexibility and toughness of PLA that are entirely, or at least partially, derived from renewable sources [18].

An interesting solution to overcome the brittleness of PLA is the use of poly(*L*,*D*-lactic acid) (PLDLA), a copolymer obtained by the polymerization of a racemic mixture of *L*-lactide and *D*-lactide enantiomers. Unlike poly(*L*-lactic acid) (denoted here by PLA), which is a semicrystalline polymer, PLDLA is an amorphous, atactic polymer with higher structural flexibility [19] and thus, higher processability as compared to PLA.

The addition of plasticizers to PLA is known to induce an increase in the mobility of the PLA chains, along with a decrease in glass transition temperature (*T_g_*) and melt viscosity, which improves the ductility and processability of PLA [20]. Lactide and oligomers of lactic acid, citrate and adipate esters, poly(ethylene glycol), poly(propylene glycol), glycerol, and glycerol derivatives are just a few bio-based plasticizing agents that have been proposed for adjusting PLA’s flexibility. However, cardanol and derivatives of cardanol [21,22], levulinic acid esters [23], maleic acid and its esters [21], and vegetable oils and their epoxidized/maleinized/acrylated/hydroxylated derivatives [24] are also plasticizers originating from renewable resources that have been studied in the last decade for enhancing PLA properties. Although many of them have proven to be extremely effective in improving the flexibility and toughness of PLA, they still exhibit some limitations [23,25,26]. For example, by employing acetylated malic acid butyl ester as a bio-based plasticizer for PLA, Park et al. [25] obtained an increase in the elongation at break of PLA from around 16 to 648%, with a simultaneous decrease in the *T_g_* from 60 °C to 38 °C. However, the increase in flexibility was accompanied by a significant decrease in the tensile strength and elastic modulus of PLA. Moreover, the plasticizer began to vaporize from the material at about 200 °C, which is not convenient, considering that neat PLA’s processing is usually conducted at 160–220 °C [27]. By the addition of 20 wt% of renewable sources-derived glycerol dilevulinate (ED) in a PLA formulation, Xuan et al. [23] reported a remarkable increase in the elongation at break of PLA to 546% (from 5% for pure PLA) and a significant decrease in the *T_g_* to 15 °C (from 59 °C for neat PLA). In another work, Zych et al. [26] reported an improvement in the elongation at break of PLA from around 2 to 368% and 783%, respectively, along with a decrease in the *T_g_* value from 71.3 °C to 51.2 °C and 47.0 °C, respectively, for PLA formulations plasticized with 10 and 40 wt% epoxidized soybean oil methyl ester (ESOME). Moreover, about 192-fold increase in the PLA toughness was achieved at a content of ESOME of 10 wt%, while above this concentration, phase separation processes were observed. Nevertheless, both ED and ESOME caused drastic decreases in the tensile strength and elastic modulus of PLA and showed an increased tendency of migration in different food simulants [23,26].

The copolymerization of *L*-lactide with different monomers emerged as another attractive technique to obtain PLA-based materials with increased flexibility and toughness [28]. In this sense, comonomers such as glycolide, ethylene glycol, ε-caprolactone, δ-valerolactone [29], β-methyl-δ-valerolactone [30], 1,5-dioxepan-2-one [31], 1,3-trimethylene carbonate [29], and ethylene carbonate [32] have been studied over time. The limitations of the copolymerization method are related to the long reaction times, high costs, difficulty in establishing and controlling the reaction conditions (reaction temperature and time, comonomer feed ratio, type of catalyst, amount of catalyst, etc.) in order to obtain copolymers with the desired properties, and the more laborious transfer of the process to an industrial scale as compared to the plasticization method [28]. Zhang et al. [33] reported the synthesis of poly(lactic acid-co-ε-caprolactone) copolymers with different flexibilities by the ROP of *L*-lactide and ε-caprolactone at 150 °C, using stannous octoate as a catalyst and varying the feed molar ratio between the two monomers and polymerization time. An elongation at break as high as 2661.3 ± 575.9% and improved toughness were obtained at a molar feed ratio of 1:1 between *L*-lactide and ε-caprolactone and a reaction time of 30 h. In another study, Xi et al. [34] obtained a flexible poly(*L*-lactide-co-trimethylene carbonate) copolymer with an elongation at break of 512% and a *T_g_* of 34.1 °C by the ROP of *L*-lactide and trimethylene carbonate at 130 °C for 72 h, using stannous octoate as catalyst and a molar ratio of 60/40 between the two monomers.

A less costly, more time-efficient, and simple technique to obtain PLA-based materials with increased flexibility and toughness is blending PLA [35] with “softer” polymers that possess lower *T_g_*, higher flexibility, and increased ductility as compared to PLA [36]. In this regard, the use of polymers entirely or partially derived [36] from renewable sources, such as starch [37], poly(butylene succinate), poly(butylene succinate-co-adipate) [38], poly(glycerol sebacate) [37], poly(butylene succinate-co-terephthalate), poly(butylene adipate-co-terephthalate) [38], etc. has been explored. An essential requirement in this technique is the existence of strong interfacial interactions, i.e., a good compatibility between the constituent polymers of the blend [39]. Poor compatibility between polymers leads to the aggregation of the particles of the more flexible polymer (the dispersed phase) in the PLA matrix, and an insufficient improvement in the elongation at break and toughness of PLA [40]. Unfortunately, most bio-based flexible polymers show poor compatibility with PLA. However, promising results were obtained by Hu et al. [41], who prepared blends using PLA and a series of self-synthesized bio-based poly(lactate/butanediol/sebacate/itaconate) (PLBSI) elastomers. An elongation at break as high as 324% and an impact strength of 35.7 kJ/m^2^ (approximately 50 and 15-fold higher than those of neat PLA) were reported at a content of PLBSI in the blend of 15 wt%. These results indicated a high compatibility between the two polymers, which was attributed to the structural similarities (lactate units, ester groups) between PLA and PLBSI. In another study, Kang synthesized a bio-sourced elastomer (BE) [42] based on sebacic acid, itaconic acid, succinic acid, 1,3-propanediol, and 1,4-butanediol as toughener for PLA. With a BE content of 11.5 vol% in the PLA/BE blend, a maximum elongation at break of 179% and a notched impact strength of 10.3 kJ/m^2^ were obtained, which are 25-fold and 4-fold higher, respectively, than those of neat PLA.

Another promising candidate for improving the flexibility and toughness of PLA may be poly(butylene sebacate) (PBSe), a bio-based and biodegradable aliphatic polyester that can be synthesized by the polycondensation of sebacic acid (Se) with 1,4-butanediol (BDO) [43]. The bio-based character of PBSe comes from the fact that both of its precursor monomers can be obtained from renewable sources, Se being produced by the alkaline pyrolysis of castor oil [44] and BDO being obtainable via the microbial fermentation of sugars from renewable sources [45]. Similar to PLA, PBSe is a biodegradable polymer in composting conditions [46]. With a *T_g_* of −29.8 °C [43], PBSe possesses a good flexibility, which could be attributed to the relatively long methylene (-CH_2_-) chain segments in its structure. The presence of ester bonds in both PBSe and PLA structures may ensure some compatibility between the two polymers, while the flexible chains of PBSe are expected to cause an increase in the flexibility of PLA.

In this work, a series of bio-based polyesters aimed to improve the ductility and toughness of PLA were synthesized by the polycondensation of sebacic acid and 1,4-butanediol, two monomers that are obtained at a commercial scale from renewable raw materials. The polycondensation reaction was carried out by varying the Se: BDO molar feed ratio, and the resulting polyesters were characterized in terms of molecular weight, polydispersity, structure, and thermal properties. Subsequently, the synthesized polyesters were employed in the preparation of PLA/polyester blends via melt compounding using different concentrations of PBSe and carefully established processing parameters. Since PBSe is bio-sourced and biodegradable, the renewability and biodegradability of PLA were not compromised. Moreover, the effects of the synthesized polyesters on the thermal properties, mechanical properties, morphology, and rheological behavior of PLA were investigated and discussed. To the best of our knowledge, this is the first time when PBSe was used as a plasticizer for tuning the ductility of PLA. The resulting PLA/PBSe blends are promising materials for the fabrication of sustainable children’s toys or other goods which require increased flexibility, in addition to other properties.

## 2. Materials and Methods

### 2.1. Materials

Polylactic acid (PLA) Ingeo 4043D, with an *L*-lactide content of 98%, an average molecular weight of 111 kDa, and a density of 1.24 g/cm^3^, was supplied by Nature Works (Blair, NE, USA). Sebacic acid (Se) (purity 99%) and titanium (IV) butoxide (TBT) (purity 97%) were purchased from Aldrich (Milwaukee, WI, USA), while 1,4-butanediol (BDO) (purity 99%) was procured from Merck Co. (Darmstadt, Germany). All chemicals were used as received without further purification.

### 2.2. Synthesis of PBSe

**Scheme 1:**
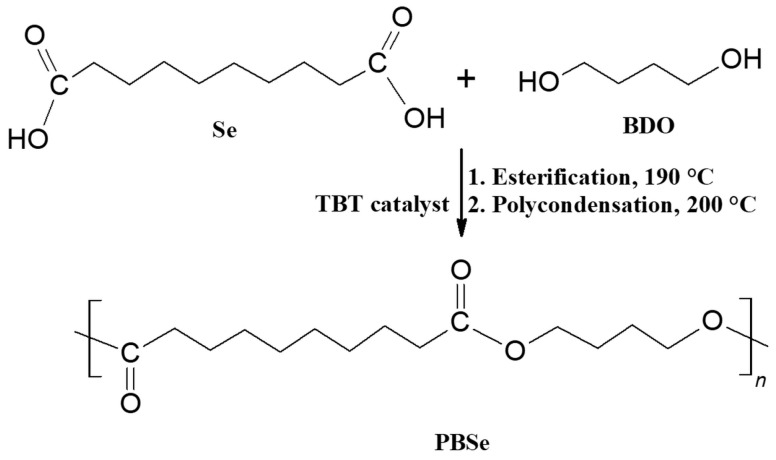
Reaction scheme for the synthesis of PBSe.

PBSe was synthesized via a two-step melt polycondensation reaction, as illustrated in Scheme 1. For this, the calculated amounts of Se and BDO were charged in a 250 mL three-neck round-bottom reaction flask equipped with a thermometer, a nitrogen inlet, and a condenser for collecting the water that results as a condensation by-product. The molar feed ratio between Se and BDO was varied so that different excesses of BDO and Se, respectively, were ensured. The amounts of Se and BDO employed in each experiment and the code name for the poly(butylene sebacate) obtained in each case are listed in Table 1. Each of the samples was denoted by “PBSey,” where y is an indicator of the ratio between the two reactants in the feed mixture, as shown in Table 1. In the first stage of the reaction, called “esterification,” the reaction mixture was heated at 190 °C for 1 h, under rigorous stirring and nitrogen atmosphere, in the absence of any catalyst. During this step, sebacic acid reacted with 1,4-butanediol, with the formation of short chain oligoesters and water as a by-product. In the second stage, called “polycondensation,” TBT (0.75 wt% relative to the Se amount) was added as a transesterification catalyst, and the reaction mixture was heated at 200 °C for 4 h, under stirring and nitrogen atmosphere. During this stage, the oligomers resulted in the “esterification stage” condensed to form polyesters with higher molecular weights. Toward the end of the reaction, a significant increase in the viscosity of the reaction mixture and hindered stirring were observed, indicating that an important increase in the molecular weight and chain length of the reaction products had taken place. At the end of the reaction, the resulting molten mass of polyesters was poured onto a glass surface to solidify. Subsequently, the obtained polyesters were used in the obtaining of PLA-based blends. Similar work concerning the synthesis of poly(butylene sebacates) by polycondensation was reported by Kim et al. [43].

All the synthesized PBSe, regardless of the molar composition of the feed mixture, were solid and tough at room temperature, having a wax-like appearance. The PBSe sample prepared using the highest excess of BDO (PBSe2) was slightly sticky as compared with the other polyesters from the series. The polyesters obtained by employing a BDO excess (PBSe1, PBSe2) exhibited a pale white color, while the ones prepared using an Se excess (PBSe3, PBSe4) were a pale cream color.

### 2.3. Preparation of PLA/PBSe Blends

PLA/PBSe blends were prepared by melt mixing PLA and PBSe in a 30 cm^3^ chamber of a Brabender mixer (Brabender GmbH & Co. KG, Duisburg, Germany), for 13 min, using carefully established temperature/rotor speed/time profiles in order to ensure a good incorporation of the PBSe in the PLA matrix. Based on repeated experimental trials, the temperature/rotor/speed/time profile was set to include three stages: (i) temperature: 165 °C; rotor speed: 100 rpm; mixing time: 2 min; (ii) 155 °C; 50 rpm; 5 min; (iii) 155 °C; 100 rpm; 5 min. These conditions ensured both a rapid melting of PLA (first stage) and a good incorporation of PBSe. The concentration of PBSe in the blends was varied in the range between 2.5 and 20 wt%. The code names and compositions of the PLA/PBSey (y being 1, 2, 3, or 4) blends are summarized in Table 2. Films of the PLA/PBSe blends having a uniform thickness of 1.00 mm (±0.03 mm) were obtained by compression-molding using a laboratory P200E platen press (Dr. Collin, Maitenbeth, Germany) at 170 °C under the following conditions: pre-heating with 0.5 MPa for 150 s, pressing at 10 MPa for 60 s, and cooling in a cooling cassette at 0.5 MPa for 60 s. A reference sample based on pristine PLA was processed under the same conditions.

### 2.4. Characterization Methods

#### 2.4.1. Fourier-Transform Infrared Spectroscopy (FTIR) Analysis

The FTIR absorbance spectra of the obtained polyesters were recorded using a Jasco FTIR 6300 instrument (JASCO Int. Co., Ltd., Tokyo, Japan). The PBSe polyesters and Se were firstly ground with KBr powder and pressed to form disc samples. Then, the FTIR data were collected between 400 and 4000 cm^−1^, with a 4 cm^−1^ resolution and 32 scans.

#### 2.4.2. Size Exclusion Chromatography (SEC-GPC)

The molecular weight distributions of the PBSe polyesters were determined using an Agilent Technologies 1200 series gel permeation chromatograph equipped with a PLgel Mixed-C column (300 × 7.5 mm) and an Agilent 1200 differential refractometer, in dimethylformamide (DMF), at 30 °C and a flow rate of 1 mL/min. The calibration was made using polystyrene standards. The PBSe samples were dissolved in HPLC grade DMF (0.1 g/5 mL) and filtered before analysis.

#### 2.4.3. Thermal Properties Analysis

Thermogravimetric analysis (TGA) was used to characterize the thermal stability of both the synthesized PBSe and the PLA/PBSe blends. TGA measurements were carried out according to ISO 11358. TA Q5000 equipment (TA Instruments Inc., New Castle, DE, USA) using nitrogen as a purge gas at a flow rate of 40 mL/min was utilized for these measurements. All the samples, polyesters, and blends were placed in platinum pans and heated from 25 to 700 °C at a heating rate of 10 °C/min. 

Modulated differential scanning calorimetry (MDSC) analysis of the PBSe synthesized polyesters was carried out using a DSC Q2000 from TA Instruments (New Castle, DE, USA) under helium flow (25 mL/min), with the base rate of 10 °C/min, amplitude of 0.8 °C/min, and a period of 30 s, as follows: cooling from 30 °C to −90 °C; heating from −90 °C to 105 °C and equilibrating for 3 min for erasing the thermal history, cooling down to −90 °C, isothermal for 3 min, and reheating to 105 °C. Instrument calibration was performed according to ASTM E 967 using indium reference.

#### 2.4.4. Scanning Electron Microscopy (SEM)

The compression molded sheets of pristine PLA and PLA/PBSe blends were fractured in liquid nitrogen and then sputter-coated (Q150R Plus, Quorum, SXE, Lewes, UK) with a 5 nm layer of gold. The surface of the fractured blends was analyzed by SEM using an environmental scanning electron microscope (ESEM-FEI Quanta 200, Eindhoven, The Netherlands) working in low vacuum, large field detector mode, at 15 kV accelerating voltage.

#### 2.4.5. Mechanical Property Analysis

Tensile tests on the PLA/PBSe blends were conducted according to ISO 527-3:2018 (applicable to films) at room temperature using an Instron 3382 universal testing machine (Norwood, MA, USA) with a load cell of 10 kN. The tensile tests were performed on rectangular samples of 60 mm × 10 mm × 1 mm (length × width × thickness), which were cut from the previously compression-molded sheets. For each sample, more than 5 specimens were measured with a crosshead speed of 2 mm/min. The average values and the standard deviations for Young’s modulus (YM), maximum tensile strength (σ_max_), and elongation at break (ε_B_) were calculated using the Bluehill 2 Software (Instron, Norwood, MA, USA).

A DMA Q800 instrument (TA Instruments, New Castle, DE, USA) was used to carry out the dynamic mechanical analysis (DMA) of pristine PLA and PLA/PBSe blends, following the guidance of ASTM D 5206 (standard test method for plastics: dynamic mechanical properties: in tension). Bar specimens of 12 mm × 6 mm × 0.5 mm (length × width × thickness), cut from the pristine PLA and PLA/PBSe blends compressed plates, were measured in multi-frequency-strain mode (tension clamp) from room temperature to 145 °C, with a heating rate of 3 °C/min and a frequency of 1 Hz. The storage modulus and the tan δ or damping factor were plotted against temperature.

#### 2.4.6. Melt Rheology Evaluation of PLA/PBSe Blends

The melt rheological properties were measured by melt flow index (MFI) using a plastometer capillary rheometer (LMI 4003 Melt Indexer, Dynisco Polymer Testing, Franklin, MA, USA). The measurements were carried out at temperature of 190 °C, using a load of 2.16 kg (ASTM 1238), in five replicates.

## 3. Results and Discussion

### 3.1. SEC-GPC Analysis

The number average molecular weight (M_n_), polydispersity index (PDI), and retention time (R_t_) of the synthesized PBSe polyesters, determined by SEC-GPC, are given in Table 1. As shown in Table 1, the polyesters obtained at a Se:BDO molar ratio of 1:1.1 showed the highest M_n_ and R_t_ values. Increasing the content of Se or BDO in the feed mixture led to lower M_n_ and R_t_ values. Indeed, the PBSe polyester samples prepared using a higher amount of Se or BDO in the polymerization substrate displayed smaller M_n_ values, which correlates with the retention time values [47]. Moreover, all the synthesized PBSe polyesters, regardless of the Se:BDO ratio, showed PDI values close to 1.

The lower M_n_ of the polymers obtained at different excesses of Se in the feed mixture can be attributed to the formation in the “esterification” stage of a larger number of oligoesters possessing strictly COOH groups at both of their ends. These oligomers did not have at their disposal sufficient oligoesters with at least one OH end group for condensation and growth in the second stage of the reaction, and thus, their chain extension was limited, and a PBSe with lower M_n_ was formed [48]. The same explanation is valid for the lower M_n_ obtained when a molar excess of 50% diol was added to the polycondensation reaction, i.e., for the PBSe2 sample. However, it should be noted that a higher M_n_ was achieved when a molar excess of 10% diol was employed, i.e., for the PBSe1 sample. This can be explained by considering the volatility of BDO, which could have evaporated, to a small extent, at the high temperatures at which the polycondensation was conducted [49]. Thus, the excess of BDO was likely lost by evaporation, and an equimolar ratio between BDO and Se may have actually remained in the polymerization substrate. An equimolar ratio between Se and BDO provides the opportunity for the formation, in the “esterification stage,” of oligoesters that possess COOH groups at both ends, OH groups at both ends, or an OH group at one end and a COOH group at the other, which increases the possibilities of chain growth in the second stage of the polycondensation reaction, leading to PBSes with superior M_n_.

According to the DSC measurements, the synthesized PBSe polyesters had a *T_g_* between −15 and −27 °C and a melting transition in the temperature range from 49 to 52 °C, depending on the molar ratio between Se and BDO (Table 3). All the polyester samples also displayed a crystallization event, which indicates them as semi-crystalline materials.

### 3.2. FTIR Analysis

**Figure 1:**
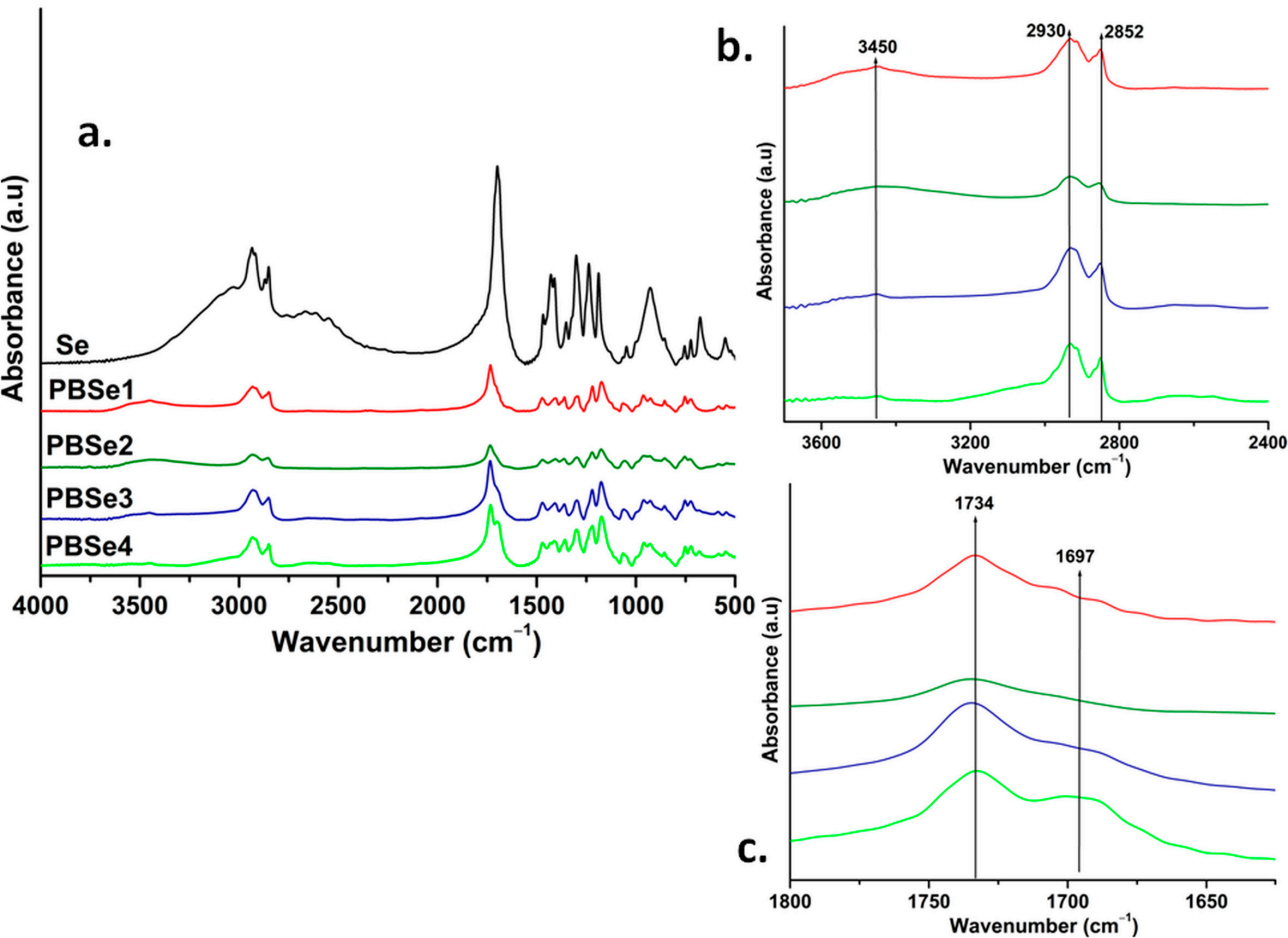
FTIR spectra of sebacic acid (Se) and PBSe polyesters (**a**) and FTIR spectra of PBSes in the range from 3600 to 2500 cm^−1^ (**b**) and 1800 to 1675 cm^−1^ (**c**).

FTIR analysis was used to characterize the molecular structure of the PBSe polyesters. Figure 1 shows the FTIR spectra of the synthesized PBSe, and the Se used as a reference. It can be observed that all PBSe spectra showed similar absorption peaks. The absorptions at 2930 cm^−1^ and 2852 cm^−1^ were assigned to the symmetrical and the antisymmetric stretching vibration of methylene groups in the polyester structures (−CH_2_), respectively [50,51]. The intensity of these two bands was greater for the PBSe samples with a higher Se content in their composition. The spectra of all PBSes also displayed a band at around 3450 cm^−1^, which can be attributed to the O-H bonds from the hydroxyl end groups of the PBSes chains which, as expected, are more intense for the PBSes obtained at an excess of BDO in the polymerization substrate [50].

An intense absorption band was seen at 1734 cm^−1^ in the FTIR spectra of all synthesized PBSe polyesters, which was associated with the stretching vibration of the carbonyl group (C=O), thus indicating the formation of ester bonds. Furthermore, the absorption at 1173 cm^−1^ confirms the presence of the C–O–C=O groups of ester bonds in the structure of all synthesized PBSe [50,51,52]. The characteristic absorption bands from the structure of Se situated at 1697, 1300, and 930 cm^−1^, which are specific to carboxylic acid groups, were not detected in the spectra of PBSe. All these results reveal the successful formation of polyester molecular structures.

### 3.3. TGA Analysis of PBSe

**Figure 2:**
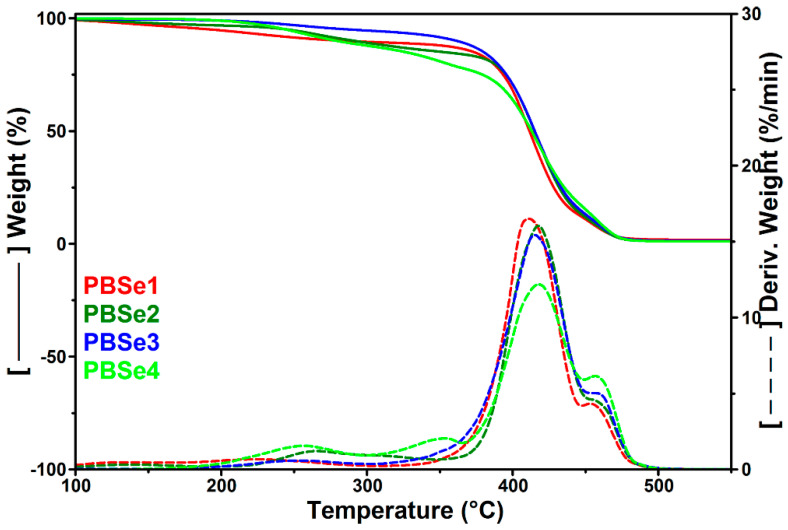
TGA and DTG curves of PBSe with different Se:BDO molar ratios.

The TGA and DTG curves of PBSe are shown in Figure 2. The related thermal parameters, i.e., the temperatures at 10% weight loss (*T_d,10%_*) and at the maximum degradation rate (*T_d,max1_*, *T_d,max2_*), the weight loss at the PLA/PBSe processing temperature of 165 °C, and the residue at 700 °C (R_700_) are listed in Table 3.

Similar two-step degradation processes were observed for all PBSe polyesters, with some small differences (Figure 2). Thus, two additional small degradation shoulders situated at around 255 °C and 349 °C were observed before the main decomposition peak in the DTG curve of the PBSe prepared with the largest excess of Se, and a broad degradation peak with a maximum at 264 °C was noted in the DTG curve of the PBSe having the highest BDO content (Figure 2). These differences could indicate the degradation at lower temperatures of the polyester with an excess of sebacic terminal moieties (PBSe4) in the first case and of the PBSe with an excess of BDO as in the case of PBSe2. Indeed, Bikiaris et al. stated that the low degradation temperatures of some aliphatic polyesters obtained from succinic acid and diols with different lengths (2, 4, 6, 8, and 10 methylene groups) could be due to the existence in these products of some oligomers with lower degradation temperatures than those of the larger macromolecules, which were difficult to remove during polycondensation, since they were prepared from diols with high boiling points, as in our case (the boiling point of BDO being at 230 °C) [53]. The previously mentioned PBSe polyester samples had the lowest *M_n_* and R_t_ values, meaning that they contain higher amounts of products with lower molecular weights, which are the first to decompose during heating [47].

The TGA results showed that the variation in the molar ratios between the components of PBSe did not significantly influence the thermal stability of the resulting polyesters: *T_d,max1_* and *T_d,max2_* values of the resulting PBSe are similar (Table 3). Similar *T_d,10%_* values were obtained for all PBSe samples, except for the PBSe3 sample, which presented better thermal stability at lower temperatures, likely because of the lower content of volatile products. Moreover, no notable differences were found between the char yields of the polyesters, a slightly higher residue being observed for PBSe1, the polyester with the highest molecular weight. In summary, considering their thermal decomposition, which took place at high temperatures ranging from 411 to 418 °C, and the small mass loss values at 165 °C (Table 3), it can be stated that all synthesized PBSe polyesters are suitable for engineering applications, in terms of processing temperature requirements.

### 3.4. TGA Analysis of PLA/PBSe Blends

**Figure 3:**
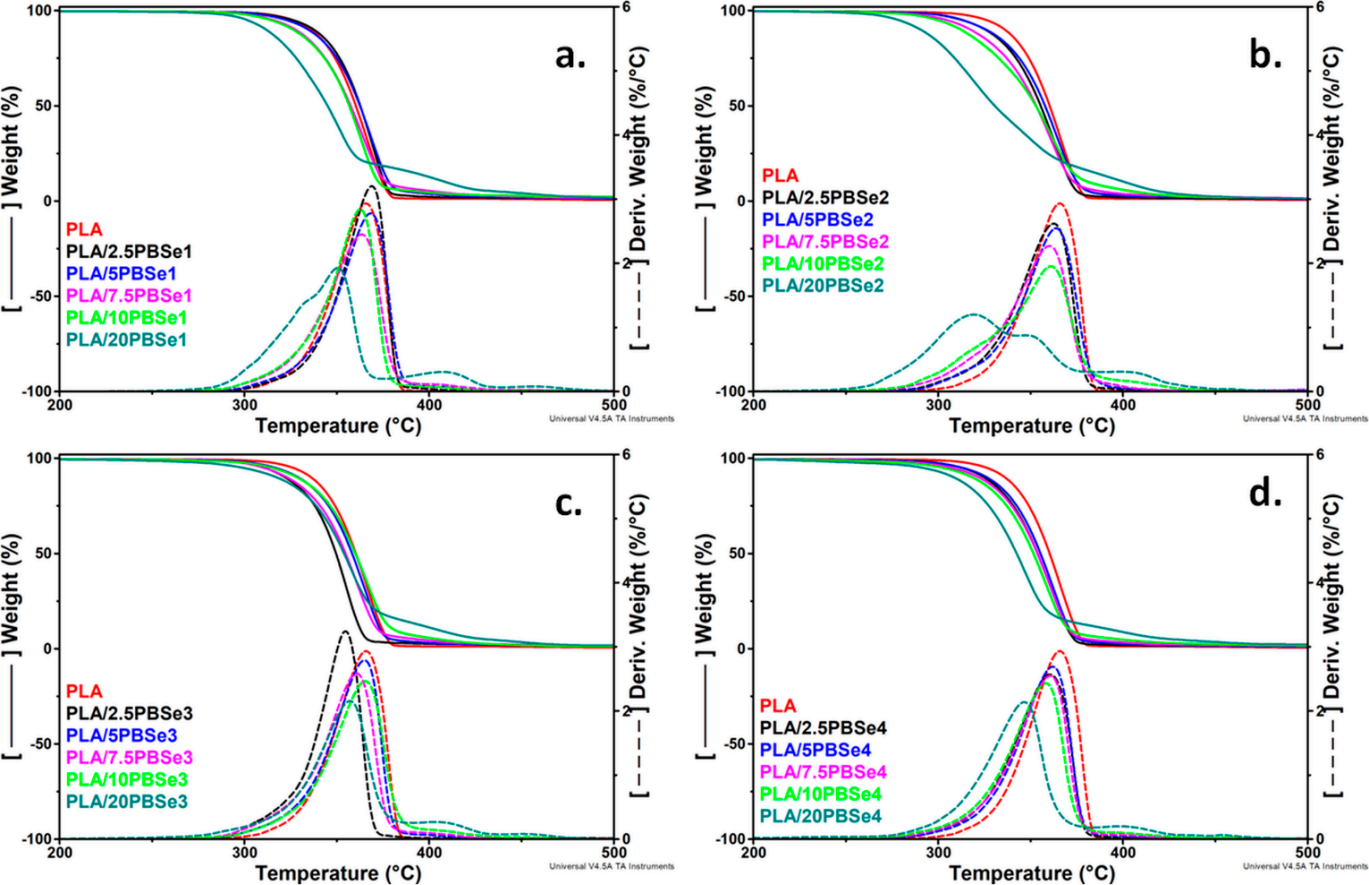
TGA/DTG curves for pristine PLA and PLA blends containing different amounts of PBSe1 (**a**), PBSe2 (**b**), PBSe3 (**c**), and PBSe4 (**d**).

TGA and DTG curves of pristine PLA and its blends with different content of PBSe polyesters (from 2.5 wt% to 20 wt%) are shown in Figure 3. The degradation temperatures at which 10 wt% mass loss occurred (*T_d_*_,*10%*_) and at the maximum degradation rate (*T_d,max_*), the weight loss at 200 °C (WL_200_), and the residue determined at 700 °C (R_700_) for the same samples are listed in Table S1.

PLA degradation occurred in a single step with a *T_d,max_* of 366 °C and a percentage of carbon residue equal to 0%, thus showing 100% degradation at 700 °C. Overall, the TGA and DTG curves of the PLA/PBSe blends showed one predominant degradation process similar to the degradation profile of pristine PLA, regardless of the PBSe composition. In general, the incorporation of PBSe polyesters promoted a small reduction in the thermal stability of the PLA blends, visible by a slight shift of the *T_d,10_*_%_ and *T_d,max_* to lower values. It is obvious that the thermal stability of the PLA blends decreases proportionally with the increase in the PBSe content. As compared to PLA, the *T_d,10_*_%_ of PLA/10PBSe1 was lower by 10 °C, that of PLA/10PBSe2 by 24 °C, that of PLA/10PBSe3 by 8 °C and that of PLA/10PBSe4 by 17 °C while the *T_d,max_* decreased only by a few degrees at this concentration of PBSes in the blends. Interestingly, the addition of low amounts (2.5 and 5 wt%) of PBSe obtained at a BDO excess of 10% led to a slight enhancement in the thermal stability of PLA (Table S1). This effect can be related to the higher M_n_ and longer molecular chains of this polyester (PBSe1), which render it harder to degrade, and to its higher melting and crystallization temperatures (Table 3).

Further, it was observed that the thermal degradation of PLA/PBSe2, containing the PBSe polyester prepared using a BDO molar excess of 50%, occurred over a broader range of temperatures. Thus, with the increase in PBSe2 content, the degradation peak of PLA decreased in intensity and was shifted to lower values, while additional small degradation shoulders appeared in the DTG curves of the PLA/PBSe2 blends. This effect was also observed in the PLA/PBSe1 blend with 20 wt% PBSe1, but at a lower intensity. In general, the PLA blends with 20 wt% PBSes had a lower thermal stability compared to PLA. This suggests that at high PBSe contents, a possible involvement of PBSe in the degradation mechanism of PLA occurred [54]. Moreover, a new small degradation shoulder was identified in the DTG curves of all PLA/PBSe blends, regardless of the PBSe type, at temperatures above 400 °C. This may be determined by the formation of cyclic or cross-linked products in polyesters at elevated temperatures [55].

However, the main degradation temperature for PLA/PBSe blends was diminished by only 5% as compared with that of pristine PLA. This means that these new materials can be processed in the molten state, without the cleavage of the polymeric chains or other decomposition processes.

### 3.5. Morphology of Fracture Surface of PLA/PBSe Blends

**Figure 4:**
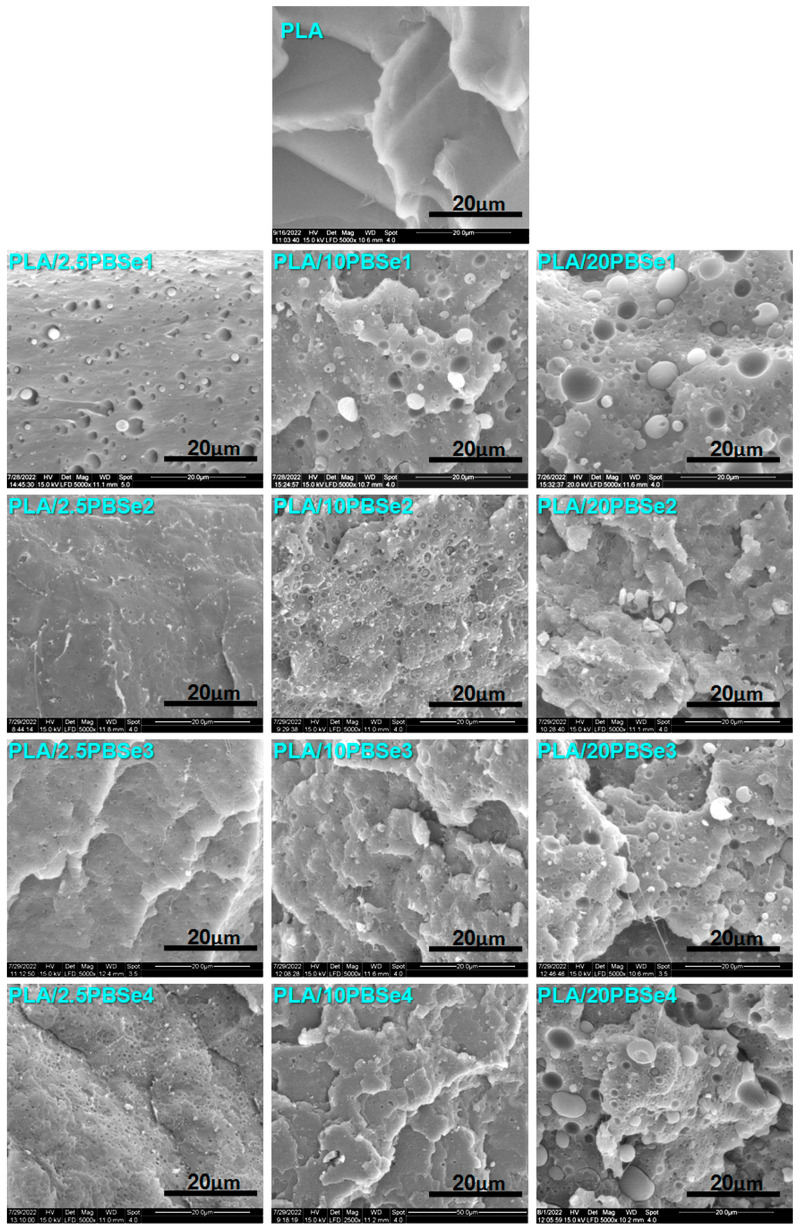
Micrographs of neat PLA and PLA blends containing different amounts of PBSe1, PBSe2, PBSe3, and PBSe4 polyesters at 5000× magnification.

The investigation of the phase morphology of the PLA/PBSe blends can provide important information regarding the microstructure/compatibility/mechanical performances relationships in these materials. The size of the dispersed PBSe particles in blends is governed by many factors, such as compatibility, viscosity match, and shear rate [42]. The micrographs of the cryo- fractured surfaces of PLA/PBSe blends at different magnifications are shown in Figures S1 and S2 and Figure 4.

A smooth surface was observed in the PLA micrograph showing its well-known brittle fracture (Figure S1). From the SEM micrographs taken at different magnifications, the following aspects were noted for the PLA/PBSe blends: (i) the PBSes form particles with spherical and irregular geometry in the blends; (ii) the PBSe particles are homogeneously dispersed in the PLA matrix, especially at low concentrations; (iii) brittle-to-ductile transition occurs with an increase in the concentration of PBSes.

The synthesized PBSe seems to have a low affinity for the PLA matrix, and they tend to bond with themselves to reduce the surface tension, which leads to the formation of spherical PBSe particles with different diameters inside the PLA matrix. This behavior was especially notable in the case of PBSe1—having a higher molecular weight—which exhibited a stronger tendency to self-assemble. Due to the limited adhesion at the PLA-PBSe interface, upon cryo-fracture, the spherical PBSe particles were pulled out causing the appearance of cavities, or even voids [42] (Figure S2 and Figure 4). Still, these features were more obvious for the PLA blends containing the PBSe1 polyester, while in all other PLA blends, this effect was visible only at the highest PBSe concentration. Indeed, the PBSe1 polyester had the highest M_n_, *T_m_*, and *T_c_* values, which influenced its dispersion in the PLA matrix.

Interestingly, at low PBSe concentrations (2.5 wt.%), the PLA blends showed a homogeneous phase microstructure, indicating a rather good compatibility between PBSes and PLA (Figure S1, 1000×). Moreover, the number average particle diameter of the dispersed PBSe particles in all the blends showed values between 0.4 and 0.7 μm, except for the PLA/PBSe1 blend, which displayed PBSe1 particles with an average diameter of 0.92 ± 0.13 μm. According to Hu et al., good compatibility between components in a binary material leads to a uniform dispersion of the dispersed phase, with small particle size distribution, as in the case of most of the PLA/PBSe blends with a small content of PBSes [52].

At a higher PBSe content (10 and 20 wt%), the fractured surface of the PLA blends showed a more ductile fracture, having more PLA matrix fibrils and a highly deformed matrix (Figure S2 and Figure 4, magnifications 2500× and 5000×). When the polyester reached the maximum concentration in the PLA blends (20 wt%), a change in the size of the PBSe particles was observed. The number average particle diameter of the dispersed PBSe particles in the PLA/PBSe blends displayed a broader range of 0.7–4 μm. A few particles having an average diameter of 14 μm were detected only in the case of blends containing the PBSe1 and PBSe4 polyesters, but only above 10 wt%. Even at higher concentration, PBSe polyesters were well dispersed in the PLA matrix (Figure 4). Noteworthy, the PBSe2 formed particles with irregular geometry, which become visible as agglomerates at 20 wt% PBSe2 in the PLA matrix (Figure 4, magnification 5000×).

**Figure 5:**
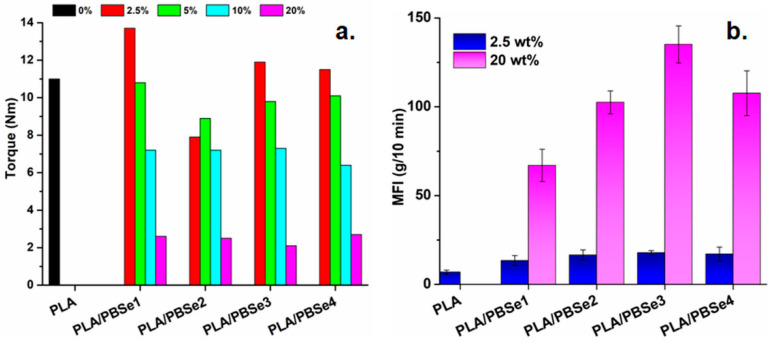
Torque (**a**) and melt flow index (**b**) values for pristine PLA and PLA/PBSe blends.

On the other hand, the SEM cryo-fractured surfaces of the PLA blends containing PBSe types 2, 3, and 4 exhibited fewer and smaller cavities and detached particles; the particles seemed more attached to the matrix, suggesting a higher interfacial adhesion and compatibility with the PLA matrix when compared with the PLA/PBSe1 blends. This may be a consequence of the lower M_n_ values of these polyesters, which may assure an enhanced solubility and dispersibility in the PLA matrix. Moreover, PLA blends with the above mentioned polyesters displayed lower torque values and higher melt flow index values (lower viscosity in the melt) as compared with the PLA blends containing the PBSe1 type polyester (Figure 5a,b).

Therefore, a higher compatibility may be presumed between the PLA and PBSe types 2, 3, and 4 due to the lower melt viscosity of these blends as compared with the PLA/PBSe1 blends. The effect of the PBSe polyesters was enhanced at higher content in the blends: a decrease in the torque values was observed with increasing PBSe content (Figure 5a), while higher MFI values were attained at higher PBSe concentrations (Figure 5b). These results also indicated a better melt processing behavior of the PLA/PBSe blends as compared to PLA. Thus, lower shear forces were involved and lower mechanical energy was consumed in the melt processing of PLA/PBSe blends than for neat PLA [56].

### 3.6. Mechanical Behavior of PLA/PBSe Blends

#### 3.6.1. Dynamic Mechanical Properties

**Figure 6:**
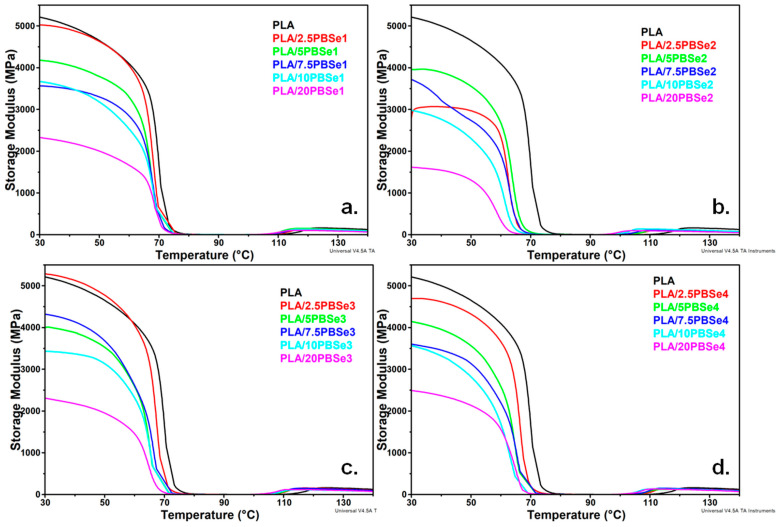
E′ curves of neat PLA and PLA blends containing different amounts of PBSe1 (**a**), PBSe2 (**b**), PBSe3 (**c**), and PBSe4 (**d**) polyesters.

**Figure 7:**
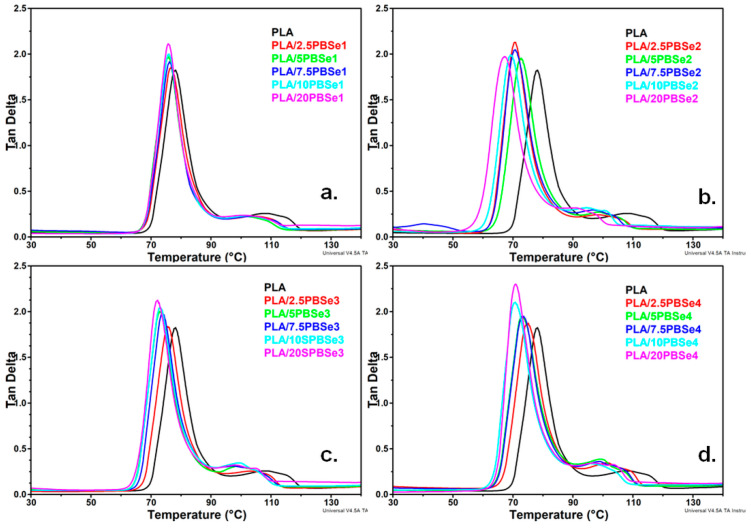
Tan δ curves of neat PLA and PLA blends containing different amounts of PBSe1 (**a**), PBSe2 (**b**), PBSe3 (**c**), and PBSe4 (**d**) polyesters.

Temperature dependence of the storage modulus (E′) and tan *δ* for pristine PLA and PLA/PBSe blends as determined from the DMA analysis are presented in Figure 6 and Figure 7. The E′ curves for pristine PLA and the PLA/PBSe blends showed a typical behavior of thermoplastic polymers, with a drastic decrease in the E′ values when approaching the glass transition temperature (*T_g_*) of PLA, and an increase starting from around 100 °C due to cold crystallization. Below *T_g_*, the E′_40_ of all PLA blends dropped gradually to lower values with an increase in the PBSes concentration, regardless of the PBSe type (Table 4, Figure 6a–d). Interestingly, cold crystallization started at lower temperatures with an increase in the PBSe content of the blend. Therefore, the addition of PBSe improved the cold crystallization ability of the PLA matrix, thus lowering its cold crystallization temperature in the resulting blends.

As shown in Figure 7, PLA exhibited a sharp tan *δ* peak at 78 °C, which corresponds to its glass transition temperature (Table 4). A depression in the *T_g_* of PLA was noticed with increasing amount of PBSe polyesters in the blends, indicating an enhancement of PLA chain segment mobility (Table 4). In particular, *T_g_* decreased from 78 °C for PLA to 76 °C for PLA/PBSe1, 67 °C for PLA/PBSe2, 72 °C for PLA/PBSe3, and 71 °C for PLA/PBSe4. The most important plasticizing effect was observed by the addition of PBSe2, and the most insignificant effect was noticed for the PLA/PBSe1 blends (Figure 7). Moreover, an increase in the height of the tan δ peak as a function of PBSe concentration was observed in all PLA blends, thus confirming the increase in segmental mobility (Table 4, Figure 7a–d).

#### 3.6.2. Tensile Properties

**Figure 8:**
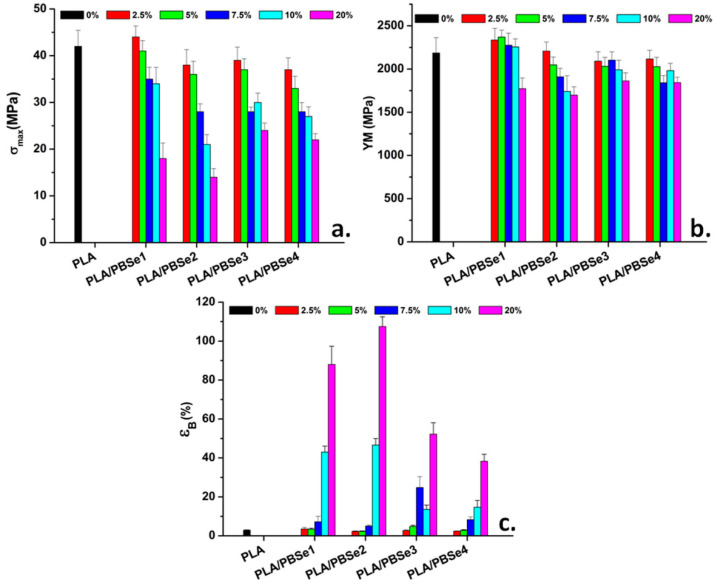
Mechanical properties determined from the tensile tests: σ_max_ (**a**), YM (**b**), and ε_B_ (**c**).

The effect of the synthesized PBSe on the mechanical properties of pristine PLA was investigated by using tensile tests. The tensile strength (σ_max_), Young’s modulus (YM), and elongation at break (ε_B_) of PLA blends containing the synthesized PBSe polyesters in different amounts are shown in Figure 8.

Pristine PLA is a stiff and brittle polymer and has a poor ductility of about 2.9%, due to extensive intermolecular forces [52,54]. The mechanical properties of PLA were strongly affected by the addition of PBSes, which showed a satisfactory affinity for the PLA matrix and manifested plasticizing effect by increasing its elongation at break (Figure 8c). This correlates with the decreasing *T_g_* values observed when increasing the PBSe content in the blends, as indicated before by the DMA results (Table 4). As observed from the ε_B_ variation, the increase in PBSe concentration promoted a proportional increase in the chain mobility of the PLA macromolecules. The PLA blends containing PBSe2 showed the highest elongation at break (107.5% for 20 wt% PBSe2), a 37-fold improvement over pristine PLA. These results were in agreement with the DMA results, which indicated that the highest decrease in the *T_g_* values occurs for the PLA/20PBSe2 blends (Table 4). However, a good improvement in the ε_B_ of the PLA blends was also recorded when PBSe1 was used as a toughener, despite the modest decrease in the *T_g_* of PLA inflicted over the entire concentration range (2.5 to 20 wt%) (Figure 8, Table 4). It is worth mentioning that this polyester had the lowest *T_g_* (−27 °C), as well as the lowest *T_c_* (Table 3), which likely contributed to an improved flexibility of the blends. The important enhancement in the PLA’s ductility is sustained by the brittle-to-ductile transition observed in the SEM images with the increase in the PBSes content in the blends, as well as by the MFI and torque results.

PBSes were able to increase the free volume between the polymer chains of PLA, resulting in higher ε_B_, while the σ_max_ and YM values decreased (Figure 8). Of all the PLA/PBSe blends, the PLA blends containing PBSe1 showed the best tensile strength and modulus, at a PBSe1 concentration of up to 10 wt% (Figure 8a,b). This can be explained by the limited miscibility between PBSe1 and PLA, which caused a lower plasticizing effect at these PBSe concentrations. As observed for other immiscible binary blends, the immiscible plasticizer may promote the crystallization of the PLA matrix through the formation of nucleation sites at their interface [52,54].

Contrarily, the PBSe2 polyester led to slightly lower σ_max_ and YM values for the PLA blends due to the presence of some irregular particle agglomerates which were detected in the SEM images, especially at higher concentrations. Moreover, the more pronounced decrease in the tensile strength of the PLA blends with PBSe2, PBSe3, or PBSe4 could be due to the miscibility of these polyesters with the PLA matrix, which weakened the interactions between the PLA macromolecules, and finally led to a decrease in the PLA’s tensile strength. These results correlated well with the decrease in the *T_g_* values of PLA in these blends, as determined by DMA.

It is important to note that although a considerable improvement in the ductility and mobility of PLA chains was attained by the addition of PBSe polyesters, the YM did not show a significant decrease. A maximum decrease in YM of only 22% was obtained at the highest content of PBSe of 20 wt% in the PLA blends (Figure 8). This is much lower than other reported results, especially when considering the remarkable improvement in the elongation at break that was attained.

## 4. Conclusions

Bio-derived poly(butylene sebacate) (PBSe) polyesters were synthesized via the direct melt polycondensation of sebacic acid and 1,4-butanediol and these were subsequently characterized. It was found that both the M_n_ and *T_g_* of the resulting PBSes can be tailored by varying the monomer ratio. FTIR results confirmed the presence of ester groups in the structure of the PBSes, while TGA showed a high thermal decomposition temperature (from 411 to 418 °C) for the prepared PBSe polyesters, accompanied by small mass loss values at 165 °C. For the first time, PBSe polyesters were used as plasticizers for tuning PLA’s ductility in concentrations from 2.5 to 20 wt%. The incorporation of PBSes into the PLA matrix led to a slight decrease in the thermal stability of PLA, which was proportional to the increase in the PBSe content in the blends. SEM results indicated a limited affinity between PBSe and the PLA matrix, which led to the formation of spherical particles of PBSe with different diameters in the PLA/PBSe blends. The PBSe particles were homogeneously dispersed in the PLA matrix, especially at low concentrations, and a brittle-to-ductile transition of the PLA matrix occurred with an increase in the PBSe concentration in the blends. An improvement in the ductility and mobility of the PLA chains was achieved by the addition of PBSe polyesters, without a drastic decrease in the Young’s modulus values. These findings demonstrated that the PBSes alone, or in blends with other biopolymers, can be promising candidates for engineering applications that require renewability in addition to good thermal and mechanical characteristics.

## Figures and Tables

**Table 1:** Code name, feed molar ratio, molecular weight, polydispersity index (PDI), and retention time (R_t_) for the synthesized PBSe samples.

Sample Code	Se: BDO Molar Ratio	M_n_ (g/mol)	PDI	R_t_ (min)
PBSe1	1:1.1	10,779± 490	1.28	1.8
PBSe2	1:1.5	7620 ± 22	1.15	1.4
PBSe3	1.1:1	7812 ± 37	1.18	1.6
PBSe4	1.5:1	6116 ± 47	1.13	1.2

**Table 2:** Code names and compositions of the prepared PLA/PBSe blends.

Sample	PLA (wt%)	PBSey (wt%)
PLA	100	0
PLA/2.5PBSey	97.5	2.5
PLA/5PBSey	95	5
PLA/7.5PBSey	92.5	7.5
PLA/10PBSey	90	10
PLA/20PBSey	80	20

**Table 3:** Thermal properties of the synthesized PBSe.

Sample	*T_d,10_*_%_, °C	*T_d,max1_*, °C	*T_d,max2_*, °C	WL_165°C_ %	R_700°_, %	*T_g_* *, °C	*T_m_* **, °C	*T_c_* ***, °C
PBSe1	284.	411	459	3. 65	1.70	−27	52	38
PBSe2	291	418	464	2.46	1.05	−23	51	37
PBSe3	362	415	463	0.61	1.22	−16	49	38
PBSe4	279	418	463	0.31	1.02	−14	47	40

* *T_g_* from DSC 1st cooling cycle. ** *T_m_* from DSC 2nd heating cycle. *** *T_c_* from DSC 2nd cooling cycle.

**Table 4:** Storage modulus (E′) at temperatures of 40 and 80 °C (E′_40_, E′_80_), damping factor (tan *δ*) values, and height.

Sample	E′_40_, MPa	E′_80_, MPa	*T_g_*, °C	tan *δ (T_g_*)
PLA	5001	13.89	78.16	1.824
PLA/2.5PBSe1	4917	9.76	76.78	1.856
PLA/5PBSe1	4056	8.62	75.83	1.968
PLA/7.5PBSe1	3490	8.21	76.26	1.910
PLA/10PBSe1	3514	8.08	75.99	1.997
PLA/20PBSe1	2208	5.82	75.80	2.111
PLA/2.5PBSe2	3065	4.76	70.65	2.128
PLA/5PBSe2	3891	5.65	72.74	1.954
PLA/7.5PBSe2	3219	4.67	70.76	2.048
PLA/10PBSe2	2765	4.24	69.38	1.988
PLA/20PBSe2	1545	2.48	67.32	1.967
PLA/2.5PBSe3	5128	9.08	75.71	1.829
PLA/5PBSe3	3881	5.72	72.97	1.998
PLA/7.5PBSe3	4135	6.29	73.84	1.987
PLA/10PBSe3	3369	5.07	73.05	2.043
PLA/20PBSe3	2175	4.36	72.18	2.125
PLA/2.5PBSe4	4617	7.40	74.91	1.864
PLA/5PBSe4	3955	4.73	73.18	1.935
PLA/7.5PBSe4	3460	5.44	73.39	1.946
PLA/10PBSe4	3320	3.46	70.78	2.100
PLA/20PBSe4	2368	3.57	70.88	2.300

## Data Availability

Not applicable.

## Supplementary Materials

The following supporting information can be downloaded at: https://www.mdpi.com/article/10.3390/polym14193998/s1, Table S1: TGA/DTG data for pristine PLA and PLA/PBSe blends; Figure S1: Micrographs of neat PLA and PLA blends containing different amounts of PBSe1, PBSe2, PBSe3 and PBSe4 polyesters at 1000× magnification.; Figure S2: Micrographs of neat PLA and PLA blends containing different amounts of PBSe1, PBSe2, PBSe3 and PBSe4 polyesters at 2500× magnification.
