# Supplementary material for: Bio-Based Poly(lactic acid)/Poly(butylene sebacate) Blends with Improved Toughness
Source: Polymers (Basel). 2022 Sep 24;14(19):3998. doi: 10.3390/polym14193998 (PMC9572606; doi:10.3390/polym14193998)
Supplement: Supplementary file 1 [file polymers-14-03998-s001.zip › polymers-1928335-supplementary.pdf]

Supplementary Material

Table S1. TGA/DTG data for pristine PLA and PLA/PBSe blends

Sample	$T_{d,10\%}$,°C	WL_{200}, %	$T_{d,max}$,°C	$R_{700°C}$, %
PLA	336	0.47	366	0.00
PLA/2.5PBSe1	338	0.39	369	1.01
PLA/5PBSe1	335	0.38	368	1.09
PLA/7.5PBSe1	328	0.35	364	1.30
PLA/10PBSe1	326	0.34	363	1.86
PLA/20PBSe1	312	0.41	351/409	1.49
PLA/2.5PBSe2	325	0.36	363	0.02
PLA/5PBSe2	326	0.47	364	0.03
PLA/7.5PBSe2	317	0.44	360	0.02
PLA/10PBSe2	312	0.33	361	0.00
PLA/20PBSe2	290	0.37	320/351/406	0.04
PLA/2.5PBSe3	322	0.47	355	0.09
PLA/5PBSe3	329	0.43	365	1.17
PLA/7.5PBSe3	323	0.45	361	1.37
PLA/10PBSe3	328	0.48	365	0.98
PLA/20PBSe3	317	0.63	357/406	1.45
PLA/2.5PBSe4	325	0.42	361	0.01
PLA/5PBSe4	326	0.44	362	0.03
PLA/7.5PBSe4	322	0.50	361	0.02
PLA/10PBSe4	319	0.54	358	0.03
PLA/20PBSe4	308	0.66	347/401	0.05

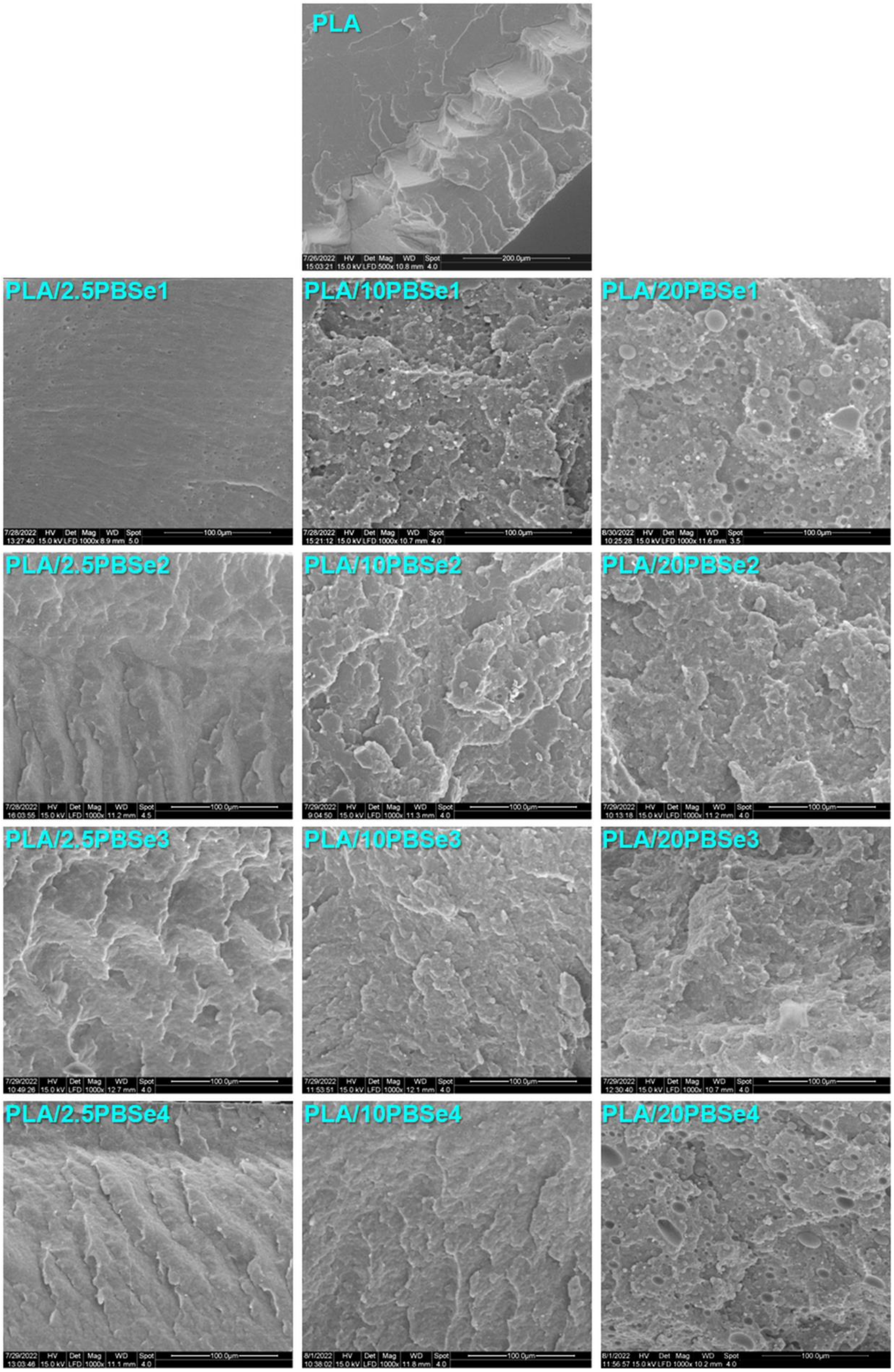

Figure S1. Micrographs of neat PLA and PLA blends containing different amounts of PBSe1, PBSe2, PBSe3 and PBSe4 polyesters at 1000× magnification.

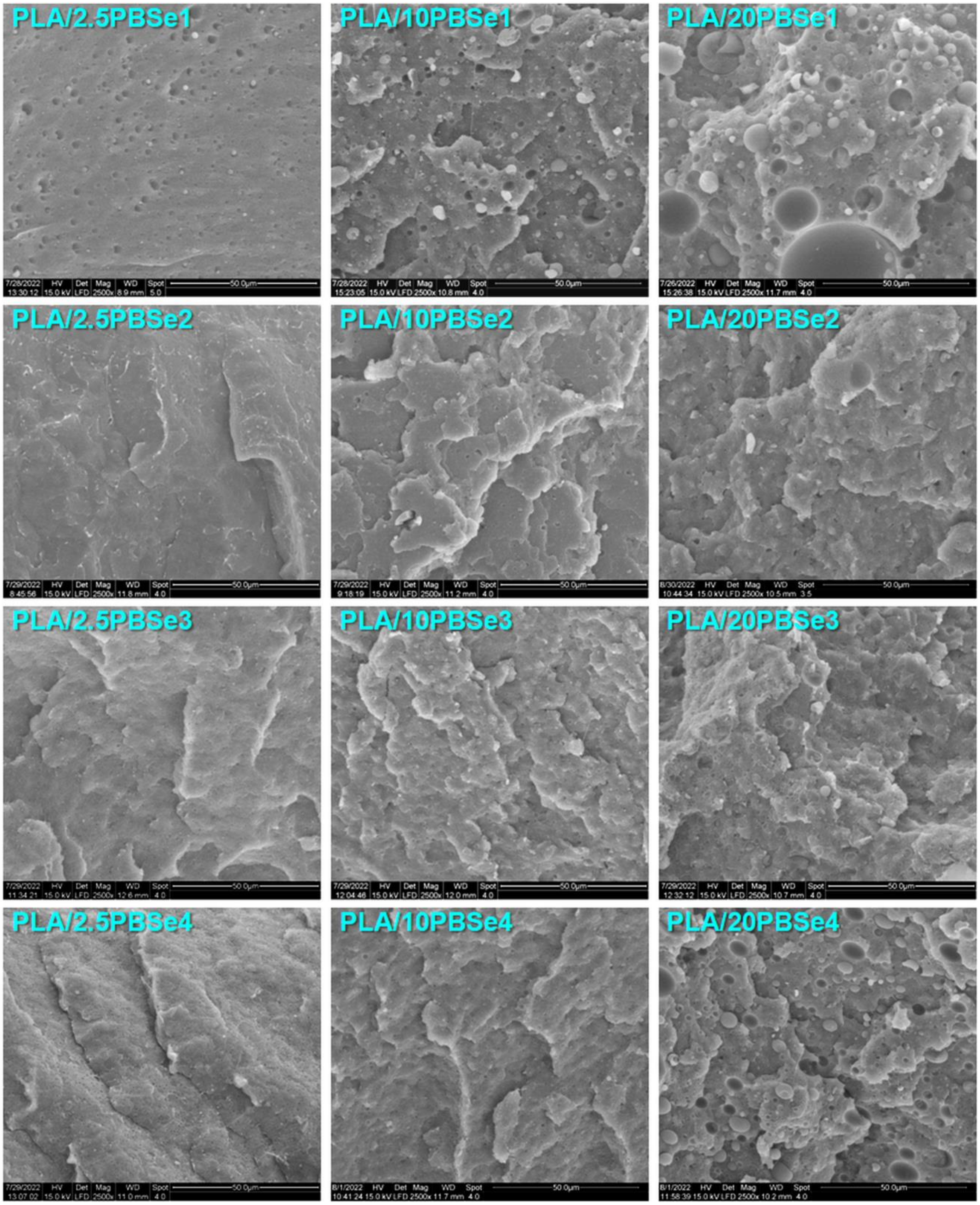

Figure S2. Micrographs of neat PLA and PLA blends containing different amounts of PBSe1, PBSe2, PBSe3 and PBSe4 polyesters at 2500× magnification.
